# The Role of Denial in Vaccine Skeptics and “Anti-vax” Blame: A Psychodynamic Approach

**DOI:** 10.3389/fpsyg.2022.886368

**Published:** 2022-07-14

**Authors:** Olivier Putois, Julie Helms

**Affiliations:** ^1^SuLiSoM UR 3071, Faculté de Psychologie, Université de Strasbourg, Strasbourg, France; ^2^Service de Psychiatrie, Santé Mentale et Addictologie, Hôpitaux Universitaires de Strasbourg, Strasbourg, France; ^3^Institut d’Immunologie et d’Hématologie, Institut Thématique Interdisciplinaire TRANSPLANTEX NG, Université de Strasbourg, Strasbourg, France; ^4^Université de Strasbourg (UNISTRA), Faculté de Médecine Hôpitaux Universitaires de Strasbourg, Service de Médecine Intensive-Réanimation, Nouvel Hôpital Civil, Strasbourg, France; ^5^INSERM (French National Institude of Health and Medical Research), UMR 1260, Regenerative Nanomedicine (RNM), FMTS, Strasbourg, France

**Keywords:** denial, vaccine skepticism, anti-vax, COVID-19, fetishism, risk perception, selfishness, self-preservation

## Abstract

In this paper, we propose to account for the blame addressed to vaccine skeptics and “anti-vax” (VS and AV) by considering their attitude as the result of the psychological mechanism of denial, understood in a psychodynamic manner. To that effect, we draw on a secondary account of our clinical experience in two hospital units (psychiatry and intensive care unit), and on openly available media material. First, we lay out how VS and AV can be understood as the result from fetishist risk denial, a specific psychological transaction with an object by which VS and AV people feel intimately protected; this object is viewed as so powerful that its protection makes the vaccine appear irrelevant. Second, we show how this mechanism can explain the specific content of the blame frequently addressed to VS and AV, who are reproached with being selfish by vaccinated people and caregivers. We contend that, contrary to common belief, they are thus blamed because they force others (and especially caregivers) to compensate their lack of self-protection and preservation, which derives from their exclusive relation to an almighty object. While such a relation accounts for the unwillingness to consider vaccination, it also explains the harshness of the blame voiced by caregivers, who feel helpless in most situations as they cannot effectively force VS and AV to take care of themselves and others.

## Introduction

One of the most frequently blamed groups during the COVID pandemics is the heterogeneous set referred to as vaccine skeptics and “anti-vax” (hereafter VS and AV). Amongst vaccinated people and caregivers, many feel that this blame is justified: their attitude increases the risk of contagion, while overburdening the healthcare system. Yet, as Bouguettaya et al. (2022) have stressed, blame in a context of pandemics affects relationships, promotes devaluation of caregivers, and prompts discrimination: it is thus necessary to account for the emergence of blame, in order to devise alternate responses to vaccine refusal. This is crucial in France where vaccine acceptance rates have been very low (Sallam, 2021).

Blame could thus be fruitfully understood and circumvented by understanding vaccine refusal. Schmitz et al. (2022) have recently explored vaccination motivation. Correspondingly, research has shown lower socio-economic status, education, distance with the government (Paul et al., 2021) and political affiliation (Fridman et al., 2021) to predict vaccine refusal. Some the most important determinants of uncertainty and unwillingness to vaccinate appear to be strong mistrust of vaccine benefit and concerns about unforeseen side effects (Paul et al., 2021).

While Goldberg (2021) has stressed the importance of a psychiatric approach to anti-vax attitudes, a specifically psychodynamic perspective hasn’t yet been explored. The goal of such an approach would be to flesh out be a non-informational, non-cognitive process underlying vaccine refusal (Hornsey et al., 2018). We thus propose to address vaccine skepticism and refusal through the psychological mechanism which we believe underlies it: denial. We believe it can shed light on the blame often addressed to VS and AV, and especially on its content (that of “being selfish”). To that effect, we draw on clinical practice in the Psychiatry and Intensive Care Units of a French University Hospital during the COVID-19 pandemics, and on media posts and declarations. Our data did not need ethical clearance, as it was a secondary account of our experiences in healthcare.

### Denial as a Psychological Determinant of Vaccine Skeptics and Anti-vax Attitudes

One empirical feature in the attitude and behavior of VS and AV people encountered in our hospital units is particularly recurrent. Generally, when discussing vaccination status, they explained that they (or their children) didn’t need the vaccine because something else was already protecting them so effectively that it made the vaccine irrelevant. This is consistent with the correlation between COVID-19 vaccination willingness and perceived vaccine effectiveness (Wake, 2021).

Some felt protected by their religious faith or spirituality; that is, by a close relationship with an almighty figure. In the Intensive Care Unit (ICU), the pious family of a deceased young man were astonished that he had died in spite of his strong faith–as though faith was a protection from contagion. A patient in the psychiatry unit said that as a healer, her contact with the energies of life protected her from catching the virus, thereby making vaccination irrelevant.

Most of these people presented no additional signs of delusional behavior or beliefs; this is not to say that their belief in a stronger protection is in itself delusional, or the sign of a delusion. Correspondingly, they knew perfectly well where to find medical information about the disease; most were well aware that many had died from it. Thus access to and knowledgeability about medically relevant information was not the explanation for their vaccine attitude, which is consistent with research showing that vaccine attitude isn’t influenced by medical information availability (Fridman et al., 2021). They had no problem acknowledging the severity of the disease, but felt they were protected from the virus by their connection with a stronger force; they behaved as though carrying a charm-laden talisman.

A psychodynamic approach to denial (Freud, 1940; Fain, 1971; Braunschweig and Fain, 1975) sheds light on such attitudes: we contend that they display a fetishist stance (Fain, 1971), which aims to enable the individual to deny that he is at risk. The word “fetish” comes from Portuguese language, and was initially used by colons referring to practices witnessed in African tribes, where a specific item was used as a protection against bad spells and dangerous encounters. The item is endowed with magical powers coming from a particular source (spirit, etc.), with which the fetish connects the individual, who becomes protected in return by the source. In a psychodynamic approach, fetishism refers to a specific psychological mechanism drawn upon by the individual presented with, or envisioning, traumatic events (harm, death, etc.) which trigger anxiety. Faced with a traumatic perspective, some individuals engage in fetishism. Fetishism is a specific psychological transaction, akin to a pact (Braunschweig and Fain, 1975 talks about a “community of denial”). Its terms are the following: if the individual unconditionally and exclusively acknowledges the power of a specific object (cause, group, deity, etc.) which presents itself as an absolute protection against harm, then the object will share with him in return some of its protective power, through a fetish that represents this power. This pact will allow the individual to deny that the initially perceived risk should be a source of anxiety. For example, in the example above, which displays a fetishist stance with respect to faith, the acknowledgment of God’s power is rewarded by His protection–a fraction of His power is granted to the individual. Engaging in fetishist denial creates a splitting in one’s mind (Freud, 1940; Fain, 1975): the risk is both initially perceived, and subsequently dismissed on grounds of the object’s acknowledged power. Thus, as opposed to “COVID-phobia” (Dilbaz et al., 2020; Nazlı et al., 2022), or “Fear of COVID” (Ahorsu et al., 2020), denial will not result in strong emotional reactions as its goal is precisely to silence the initial perception of anxiety which caused them.

It should thus be borne in mind that:

1. The psychological function of the fetish is to protect the individual against the anxiety triggered by the perception of potential harm or risk (contagion, death, etc.), by enabling the denial of this perception. [Denial is a defense mechanism–on the relevance of defense mechanisms (cf., Malan, 1982; Plutchik, 1995)];
2. The acknowledgment of the object’s power needs to be exclusive and without restriction. Ignoring this condition will lift off the object’s protection.

Importantly, the object which appears powerful or almighty is referred to in such abstract terms in psychodynamic theory because, as mentioned earlier, it doesn’t have to resemble a person–as could be the case with, say, an object of worship. Truth, as an object of knowledge or conviction, can be the object to which the individual believes he is intimately connected. Being convinced of this connection, he feels he can recognize as evidence of his belief signs overlooked by people who lack his conviction. Such signs function like fetishes, assuring him that his belief (“I am protected”) is true, and that his knowledge helps him see through dubious discourse. The fetishist relation to the object thus feeds denial by legitimizing the ignorance of facts that run contrary to the individual’s belief (such as “the virus exists, and it has killed X thousand people”). The individual engaged in fetishism takes people who hold these facts true to simply lack his privileged access to truth, which makes those facts appear dubious in contrast. In cases when the object is truth, almightiness takes the form of infallibility. Fetishism thus provides a potential psychological mechanism underlying many versions of explicitly “anti-vax” conspiracy theory speech, which frequently displays a conviction of absolute certainty; it also accounts for the oft-highlighted connection (Poupart and Bouscail, 2021), and even prediction, of VS or AV attitudes in the presence of prior adhesion to conspiracy theories (Al-Jayyousi et al., 2021; Nazlı et al., 2022).

This psychodynamic approach to denial sheds light on the claim, voiced by many VS and AV (even on their deathbed!), according to which the vaccine isn’t safe enough. At first, it sounds paradoxical: statistically speaking, refusing vaccination is much more risky, in spite of the very rare potential secondary effects of the vaccine (upon which VS and AV are often well-versed). But this benefit-risk ratio approach misses the point of the fetishist attitude, which is to enable the denial of any risk of contagion and its consequences. Considering vaccination entails that one has acknowledged the risk of contagion, and foregone the belief in an almighty protection instead of denying the risk and the subsequent need for protection. Therefore from a fetishist standpoint, considering vaccination triggers an anxiety specifically associated with the absence of an almighty protection: it is this anxiety which the fetishist seeks to deny by relying on his fetish.

Understanding the VS and AV attitude as a fetishist choice enabling the denial of COVID-19-related risks could account for the content of lots of the blame directed toward the VS and AV: they are often reproached with being selfish.

### Vaccine Skeptics and Anti-vax Fetishist Risk Denial Accounts for the Social Blame of Selfishness

A psychodynamic approach allows to understand the blame of selfishness as an effect of the VS and AV fetishist risk denial on vaccinated people, and especially caregivers.

A brief examination of samples of empirical material, such as media coverage (including blogs, op-ed columns, etc.), shows that VS and AV are quite often blamed with being selfish. French writer and blogger Sagalovitsch chose to name a 2021 Slate blog post “The selfishness of non-vaccinated people will long be remembered” (Sagalovitsch, 2021). British TV host Piers Morgan went for a slanderous Twitter comment: anti-vaxxers are “selfish pr*cks” (Evans, 2021). Even always-diplomat Spanish tennis champion R. Nadal said that AV seem “a bit selfish” (Kershaw, 2021). This blame always follows the same initial statement: they only think about themselves (Deray, 2021; Evans, 2021; Sagalovitsch, 2021), in that they do not seem to realize that their behavior puts others at risk.

Their behavior was deemed selfish for another reason. Past the contagion stage, COVID-19 patients prevented many vaccinated people in need of medical care from accessing it, either in specific hospital services re-allocated to COVID patients (e.g., neurology units becoming temporary COVID units) or in ICUs, where COVID patients had an almost systematic priority over other patients. As a consequence, lots of vaccinated people whose medical care was hindered by the pandemics considered that VS and AV should face the consequences, for example, with direct financial penalties (Green, 2022). It is this perceived lack of responsibility for their non-vaccination that triggered the blame of selfishness amongst vaccinated people: in essence, VS and AV were experienced to behave in a non-reciprocal, unfair manner.

This perception of selfishness is echoed by doctors, who frequently view VS and AV as selfish and irresponsible. Deray calls them a symptom of a disease of selfishness (Deray, 2021). French diabetologist Grimaldi has stated that VS and AV should be consistent with their vaccine refusal, and state in their advance directives whether they wish to be medically revived in case of severe forms of COVID-19 (Grimaldi, 2022). Both stressed that VS and AV represent a threat to social justice and fairness, by forcing to prioritize which patients should taken care of first, especially in ICUs. In spite of official information displayed at the beginning of the pandemics, COVID-19 patients whose condition worsened and required intensive care had a *de facto* priority over patients requiring ICU admission for other reasons. They would also have a longer ICU stay (at least a few weeks). Patient selection became a pressing concern (Lecouvé and Zagdoun, 2022), and relations between hospital units became more tense: ICUs had to refuse many admissions because of a COVID-19 overload. In this context, ICU caretakers have often said that, in spite of the Hippocratic Oath, it was very hard for them not to perceive VS and AV as selfish: not only do they ask for the same medical care as people who do get a vaccine (while they don’t)–they also have ICU priority over vaccinated people in need of care for other reasons, when their condition worsens because of a COVID infection.

Additionally, at a time where the medical caretaking system was close to breaking point, imposing an extra burden on it was perceived in a particularly negative manner by both caretakers and vaccinated people in general, with the latter publicly expressing a deep identification and gratitude to the former.

While “media framing” of the blame is a reality (Court et al., 2021; Bouguettaya et al., 2022), it is the inconsistency of VS and AV that vaccinated people and doctors put forward when explaining the blame of selfishness. They perceive VS and AV to rely heavily on the responsibility of vaccinated people to protect themselves and others, while at the same time denying the relevance of the vaccine. It’s as though they said to vaccinated people “if others are doing it, why should I”?

A psychodynamic standpoint on denial can account for this perceived inconsistency, which is at the root of the blame of selfishness. Contrary to what vaccinated people believe, a person engaged in fetishistic denial does not avoid vaccination because they intimately know or hope that, in the end, they will be taken care of by others. This would entail that the fetishist does not really believe in the almightiness of their object–that is, in its absolute protection. It is quite the contrary: fetishists feel so deeply bound to their object that they genuinely believe it fully protects them. Hence their surprise when being contaminated, and their reactions to the care provided by ICU teams: they often say that it is, e.g., their belief that saved them, not the doctors; or that they see no reason to get a vaccine, even after their stay in the ICU.

While this attitude is more consistent than vaccinated people and caretakers believe, it also shows that VS and AV are not selfish, in the usual sense of the term–i.e., egoistically thinking of their own interests first (safety, etc.), or anticipating subsequent external help. On the contrary, the main effect of the fetishist denial used to avoid anxiety is a perverse effect, of which they are the first victim: their prior acknowledgment of the object’s almightiness effectively put them at risk of contagion, while preventing them to realize it (doing so would question its unquestionable almightiness). In other words, a direct implication of fetishist denial is the lack of any action ensuring effective self-protection by means external to the almighty object (cf. part 1); this is shown in the post-ICU above statement that there still is no reason to get a vaccine. If the fetishist conviction is that the object requires display of belief in exchange for protection, then they will engage in effective ritual practices; but they will do nothing referring to another source of protection. While this attitude does result in exposing third parties to contagion and adds constraints to the healthcare system, it is essential to understand that the person engaged in fetishist denial is the first potential victim of his effective lack of self-preservation.

This understanding rules out blaming VS and AV for being selfish in the usual sense of the term, but it accounts for the blame of selfishness voiced by vaccinated people. The fetishistic lack of self-preservation out of the perimeter of the requirements of the pact with the object forces vaccinated people and caregivers to decide whether or not to compensate this lack by effectively protecting the VS and AV, when faced with their risk-taking behavior–or to partake in their denial of the actual risk. We believe that the blame of selfishness is a psychological effect of the VS and AV’s lack of self-preservation and unwillingness to protect themselves, on the vaccinated people and the caregivers–who are engaged in the protection of themselves and others. VS and AV are felt to be selfish because their risk-taking attitude forces others to decide whether to care for them, while displaying an open disbelief in medical protection (which differs from that of their specific object).

The harsh tone of the blame of selfishness could come from the helplessness of vaccinated people and caregivers. While such risk-taking behavior forces them to decide whether to compensate the lack of self-preservation, they are put in a position of double-bind (Bateson et al., 1956) or paradoxical injunction (Racamier, 1973; Anzieu, 1975): it is neither in their power nor in their rights to enforce vaccination (at least in France). And since caregivers in such situations obviously cannot either, for ethical reasons, enforce vaccination by threatening to condition access to care, they are left without any external means of pressure to steer VS and AV toward a safer behavior [In this light, Grimaldi’s (2022) request for explicit advance directives can be understood as a reaction to this helplessness].

## Conclusion

Blame isn’t the solution to address VS and AV rhetoric and concerns. The WHO has underlined the need to deconstruct the strategy of vocal vaccine deniers when facing them, in particular by telling the truth and not denying the limits of medical knowledge and care (World Health Organization, Regional Office for Europe, 2017).

We believe that the above considerations could contribute to interactions between caregivers and VS and AV patients, and to social interactions during a pandemics. By specifying the type of anxiety against which VS and AV want to protect themselves at the individual level, this research can help devise non-stigmatizing, blame-free responses at the institutional level. It could thus contribute to psychodynamic approaches to health policy and implementation which address how to respond to social anxiety on public health issues (Walsh et al., 2016).

To that effect, our psychodynamic hypothesis regarding the origin of the blame in individual fetishist denial (which we believe is partly confirmed by the blame of selfishness) should be tested within a more systematic, qualitative empirical research. The main question of this research would be: what individual factors trigger denial in the context of vaccination in certain people, but not in others–in both one’s life history and one’s actual environment? This research could provide different types of life trajectories of VS and AV, combining individual, social, and political (Ward et al., 2020) factors into typical profiles of denial.

## Author Contributions

OP conceived the manuscript structure, devised the argument, provided the clinical material, and wrote the manuscript. JH contributed to the manuscript structure and to the argument and provided the clinical material. Both authors contributed to the article and approved the submitted version.

## Conflict of Interest

The authors declare that the research was conducted in the absence of any commercial or financial relationships that could be construed as a potential conflict of interest.

## Publisher’s Note

All claims expressed in this article are solely those of the authors and do not necessarily represent those of their affiliated organizations, or those of the publisher, the editors and the reviewers. Any product that may be evaluated in this article, or claim that may be made by its manufacturer, is not guaranteed or endorsed by the publisher.
